# Physical disabilities caused by leprosy in 100 million cohort in Brazil

**DOI:** 10.1186/s12879-021-05846-w

**Published:** 2021-03-22

**Authors:** Mauro Niskier Sanchez, Joilda Silva Nery, Júlia Moreira Pescarini, André Alves Mendes, Maria Yury Ichihara, Camila Silveira Silva Teixeira, Maria Lúcia Fernandes Penna, Liam Smeeth, Laura Cunha Rodrigues, Maurício Lima Barreto, Elizabeth B. Brickley, Gerson Oliveira Penna

**Affiliations:** 1 Núcleo de Medicina Tropical, Universidade de Brasília, Avenida L3 Norte, s/n°, Campus Universitário Darcy Ribeiro, Gleba A, Brasília, Distrito Federal CEP 70297-400 Brazil; 2 Centro de Integração de Dados e Conhecimentos para Saúde (Cidacs), Fundação Oswaldo Cruz, Rua Mundo, s/n° Parque Tecnológico da Bahia – Trobogy, Salvador, CEP 41745-715 Brazil; 3 Instituto de Saúde Coletiva, Universidade Federal da Bahia, Rua Basílio da Gama, s/n° - Canela, Salvador, Bahia CEP 40110-040 Brazil; 4 Departamento de Estatística, Universidade Federal Bahia, Rua Barão de Jeremoabo, s/n° - Ondina, Salvador, Bahia CEP 40170-115 Brazil; 5 Departamento de Epidemiologia e Bioestatística, Universidade Federal Fluminense, Bloco do Hospital Universitário Antônio Pedro (Huap) – 3° andar, Rua Marquês do Paraná, 303, Centro, Niterói, Rio de Janeiro, CEP 24030-210 Brazil; 6 Department of Non-Communicable Disease Epidemiology, London School of Hygiene & Tropical Medicine, Keppel Street, London, WC1E 7HT UK; 7 Health Data Research (HDR), London, UK; 8 Department of Infectious Disease Epidemiology, London School of Hygiene & Tropical Medicine, Keppel Street, London, WC1E 7HT UK; 9 Escola Fiocruz de Governo, Fiocruz Brasília. Avenida L3 Norte, s/n°, Campus Universitário Darcy Ribeiro, Gleba A, Brasília, Distrito Federal CEP 70904-130 Brazil

**Keywords:** Physical disabilities, Leprosy, Socioeconomic factor, Brazil

## Abstract

**Background:**

Leprosy continues to be an important cause of physical disability in endemic countries such as Brazil. Knowledge of determinants of these events may lead to better control measures and targeted interventions to mitigate its impact on affected individuals. This study investigated such factors among the most vulnerable portion of the Brazilian population.

**Methods:**

A large cohort was built from secondary data originated from a national registry of applicants to social benefit programs, covering the period 2001–2015, including over 114 million individuals. Data were linked to the leprosy notification system utilizing data from 2007 until 2014. Descriptive and bivariate analyses lead to a multivariate analysis using a multinomial logistic regression model with cluster-robust standard errors. Associations were reported as Odds Ratios with their respective 95% confidence intervals.

**Results:**

Among the original cohort members 21,565 new leprosy cases were identified between 2007 and 2014. Most of the cases (63.1%) had grade zero disability. Grades 1 and 2 represented 21 and 6%, respectively. Factors associated with increasing odds of grades 1 and 2 disability were age over 15 years old (ORs 2.39 and 1.95, respectively), less schooling (with a clear dose response effect) and being a multibacillary patient (ORs 3.5 and 8.22). Protective factors for both grades were being female (ORs 0.81 and 0.61) and living in a high incidence municipality (ORs 0.85 and 0.67).

**Conclusions:**

The findings suggest that the developing of physical disabilities remains a public health problem which increases the burden of leprosy, mainly for those with severe clinical features and worse socioeconomic conditions. Early diagnosis is paramount to decrease the incidence of leprosy-related disability and our study points to the need for strengthening control actions in non-endemic areas in Brazil, where cases may be missed when presented at early stages in disease. Both actions are needed, to benefit patients and to achieve the WHO goal in reducing physical disabilities among new cases of leprosy.

## Background

Chronic infections with *Mycobacterium leprae* have the potential to cause lasting nerve damage and physical disabilities [1, 2]. Among patients with leprosy, physical disabilities arise as a result of late diagnosis and/or insufficient treatments. The incidence of leprosy-related disabilities among newly detected cases is, therefore, an important indicator of gaps in population-level leprosy control strategies. Leprosy cases are classified as: Grade 0 disability (G0D), when muscle strength and sensitivity of these segments are preserved; Grade 1 (G1D), when there are decreased muscle strength and/or decreased sensitivity; and Grade 2 (G2D), when there are visible deformities in the hands, feet, and/or eyes [3, 4].

As part of the 2016–2020 Global Leprosy Strategy, the World Health Organization (WHO) has set a target of reducing the rate of newly diagnosed leprosy patients with G2D to less than 1 per million population [4]. In Brazil, a country with a high leprosy new case detection rate (13.7/100,000 population in 2018), the National Leprosy Disease Program has similarly prioritized reducing the rate of diagnosis with G2D as a primary goal. From 2012 to 2016, the mean rate of leprosy new case detection with G2D in Brazil was 10.5 per 1 million inhabitants, with an average of 2042 people diagnosed annually with leprosy-related G2D in this period [5]. In the last decades, Brazil has adopted extensive public health measures to improve the assessment and prevention of leprosy-related physical disabilities [6]. Nevertheless, a systematic review conducted by Vieira et al. (2018) [7], indicates that the proportion of leprosy cases presenting disability among children < 15 years remains high in Brazil, reflecting active transmission and challenges for case detection.

Although there have been large-scale studies in Brazil studying the social determinants of leprosy incidence and treatment default [8, 9], risk factors for leprosy-associated disability at the time of diagnosis remain scarcely investigated. In Brazil, there are problems related to underdiagnosis and underreporting of new cases of leprosy, which have had a major impact on the ability to plan control activities for the disease. In addition, primary health services face difficulties in monitoring patients after completing treatment and monitoring disabilities. Using nationwide linked data from the 100 Million Brazilian Cohort, this study used large-scale data to identify risk factors for having leprosy-related physical disabilities at the time of diagnosis.

## Methods

### Study design and data source

The 100 Million Brazilian Cohort [10, 11] was built by linking health and administrative records of individuals registered in the *Cadastro Único para Programas Sociais* (CadÚnico), a national registry for social assistance programs in the country. This database was created at the Centro de Integração de Dados e Conhecimentos para Saúde at Oswaldo Cruz Foundation (Cidacs, Salvador, Bahia, Brazil) and is part of the Center’s mission to evaluate the impact of social determinants and policies on health. The cohort includes administrative records from over 114 million individuals who applied for social assistance between 2001 and 2015.

As previously described [8, 9, 12, 13] the data from the 100 Million Brazilian Cohort was then linked to leprosy notification records in the national notifiable disease system, *Sistema de Informação de Agravos de Notificação*, SINAN-leprosy.

### Settings and participants

The study population for this investigation included members of the 100 Million Brazilian Cohort followed from January 1st 2007 until December 31st 2014. Cohort members were excluded if they: (i) were diagnosed with leprosy prior to registration in CadÚnico, (ii) belonged to family units with no member aged over 15 years (i.e., children registered separately from their families), (iii) had less than 1 day of follow-up, (iv) were relapsed leprosy cases or (v) did not have information on grade of disability at diagnosis.

### Outcome and exposures

For this study, the primary outcome was the detection of physical disabilities caused by leprosy, classified as grade 0 (G0D), grade 1 (G1D) or grade 2 (G2D).

Exposure variables were related to individual socioeconomic indicators (i.e., sex, age, self-identified race/ethnicity, literacy, schooling, and employment status) and household living conditions (i.e., household density, housing materials, water source, electricity source, sewage disposal, and waste disposal). For individuals under 18 years old, education and employment were reported as the education level and employment status of the head of the family (here defined as the oldest member of the family).

Geographic exposures included Brazilian region of residence, urbanicity (urban or rural), and residence in a ‘high-burden cluster’. The definition of clusters of higher incidence was the one used by Penna et al. (2009) [14], based on epidemiological data of Brazil from 1980 until 2007. These clusters were defined as 29 spatial clusters comprising 789 municipalities and were devised to facilitate decision-making for leprosy control across the country. Although these were defined more than 10 years ago, a recent study [15] analyzed the spatial distribution of leprosy in selected endemic regions of the country comparing the periods 2001–2003 versus 2010–2012 and concluded that there is significant overlap of clusters comparing both time periods.

Clinical exposures included the operational classification of leprosy (i.e., paucibacillary or multibacillary [PB or MB]) and the number of skin lesions.

### Statistical analysis

Descriptive analysis was performed to assess the distribution of the independent variables, followed by bivariate analysis with the outcome (presence of any degree of disability) to assess the strength of association between independent variables and grade of disability at diagnosis. Those with a *p*-value less than 0.1 were considered eligible for the multivariate model.

For the multivariate analysis, a multinomial logistic regression model with cluster-robust standard errors (i.e., accounting for familial clustering of covariates) to estimate the adjusted odds ratios (OR) was used, with grade zero disability cases used as the reference category. The adjusted ORs will therefore represent the odds of the outcome (G1D or G2D versus grade zero) associated with that particular category of the independent variable compared to the reference category for the same variable.

All analyses were performed using Stata, version 15.0 (Stata Corp LLC, College Station, Texas, USA).

### Ethics

This study was performed under the international (Helsinki), Brazilian and United Kingdom research regulations and was approved by three Ethics Committees of Research: (i) University of Brasília (1.822.125), (ii) Instituto Gonçalo Muniz – Fiocruz (1.612.302) and (iii) London School of Hygiene and Tropical Medicine’s Research Committee (10580–1).

### Role of funding source

This study was funded by The Medical Research Council (MR/N017250/1), Conselho Nacional das Fundações Estaduais de Amparo à Pesquisa / Economic and Social Research Council / Medical Research Council / Biotechnology and Biological Sciences Research Council / Conselho Nacional de Desenvolvimento Científico e Tecnológico / Fundação de Apoio à Pesquisa do Distrito Federal (CONFAP/ESRC/MRC/BBSRC/CNPq/FAPDF) – Doenças Negligenciadas, (Number 193.000.008/2016), and the Wellcome Trust (202.912/B/16/Z). The funders of the study had no role in the design, data collection, analysis, interpretation, or writing the article.

## Results

**Fig. 1:**
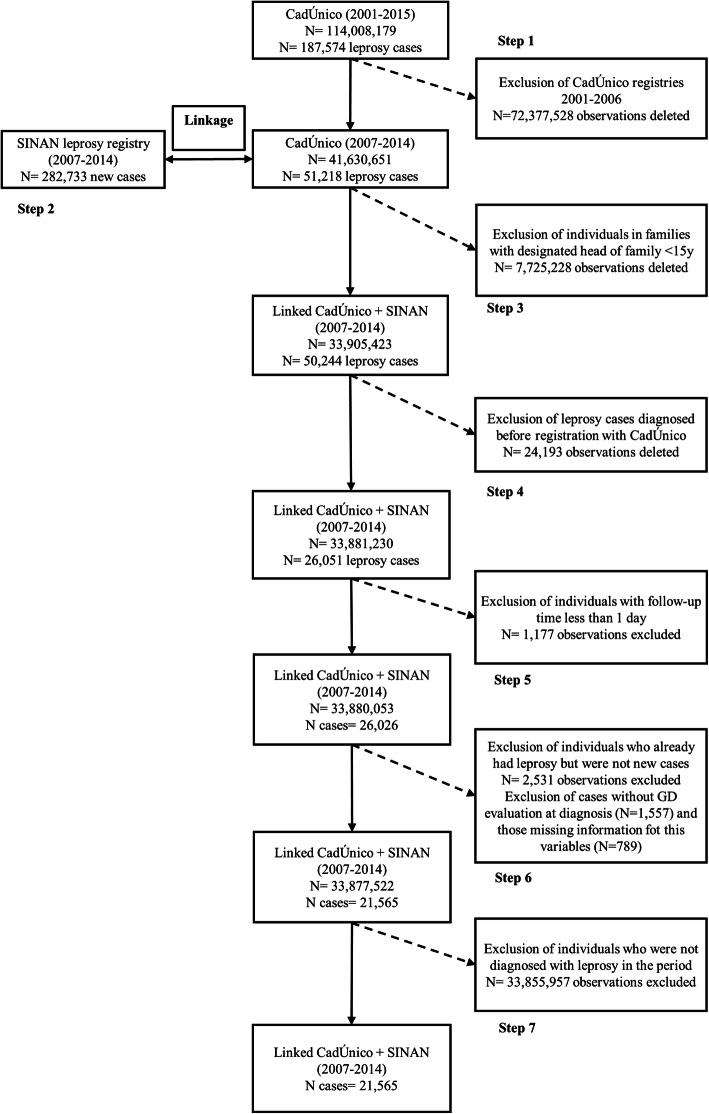
Study population selection flowchart from the 100 Million Brazilian Cohort

The study population included 21,565 new leprosy cases detected in Brazil between 2007 to 2014 (Fig. 1). At the time of diagnosis, 15,095 (63.1%) cases had G0D, while grades 1 and 2 represented 21% (5026) and 6% (1444), respectively. In the multivariate model, 16,376 cases were included, as missing values for some variables prevented a number of cases from being included.

Newly detected leprosy cases had a mean age of 37.6 years old (SD 19.5), varying by the grade of disability (G0D 34.9; G1D 43.4; G2D 45.7) (Table 1). Overall, 49.6% of the leprosy cases were female, 72.1% was identified as mixed race (“pardo”), 79.3% were literate and 60.7% had up to 5 years of education, and 50.9% were unemployed or unemployed but currently studying. Although 81.8% earned up to 0.5 minimum wage, 11.3% reported no source of income. Most of the leprosy cases lived in urban areas (79.5%) and in the Northeast (40.4%) and the North (23.6%) regions. The greater proportion of the cases lived in municipalities that belonged to the epidemiologically-defined high incidence clusters (63.8%). Also, 69.3% of the cases lived in brick or cement-made dwellings, with publicly provided water, garbage collection and electricity. However, 67% of them reported using a homemade tank as a sewage disposal system. There were more MB cases than PB (59.2% vs 40.8%) at time of diagnosis (Table 1).

**Table 1:** Characteristics of leprosy cases evaluated for physical disabilities. The 100 Million Brazilian Cohort, 2007–2014

Variables	Physical Disabilities			Total
	Grade 0	Grade 1	Grade 2	
	(N = 15,095)	(N = 5,026)	(N = 1,444)	
	n(%)	n(%)	n(%)	n(%)
**Individual characteristics**				
Age (Mean [SD])	34.9 (19.2)	43.4 (18.6)	45.7 (19.0)	37.6 (19.5)
Sex				
Male	6,984 (46.3)	2,915 (58.0)	965 (66.8)	10,864 (50.4)
Female	8,111 (53.7)	2,111 (42.0)	479 (33.2)	10,701 (49.6)
Ethnicity				
White	2,645 (17.9)	1,076 (21.8)	330 (23.2)	4,051 (19.2)
Black	1,178 (8.0)	412 (8.4)	132 (9.3)	1,722 (8.1)
Asian	49 (0.3)	19 (0.4)	1 (0.1)	69 (0.3)
Mixed (brown)	10,879 (73.5)	3,406 (69.0)	956 (67.3)	15,241 (72.1)
Indigenous	48 (0.3)	20 (0.4)	1 (0.1)	69 (0.3)
Ignored/Missing				455 (0.02)^a^
Literacy				
Yes	12,182 (81.5)	3,714 (74.6)	1,028 (71.6)	16,924 (79.3)
No	2,760 (18.5)	1,262 (25.4)	407 (28.4)	4,429 (20.7)
Ignored/Missing				234 (0.01)^a^
Schooling				
No education/Pre-school	2,266 (17.0)	1,060 (23.3)	362 (26.8)	3,688 (19.2)
Primary School (1–5 years)	5,346 (40.1)	2,034 (44.6)	595 (44.1)	7,975 (41.5)
High School (6–9 years)	3,981 (29.9)	1,101 (24.1)	302 (22.4)	5,384 (28.0)
Senior High School (10–12 years)	1,677 (12.6)	350 (7.7)	89 (6.6)	2,116 (11.0)
Higher Education (≥12 years)	48 (0.4)	14 (0.3)	2 (0.2)	64 (0.3)
Ignored/Missing				2,566 (0.1)^a^
Work condition				
Employed	6,801 (50.6)	2,073 (46.1)	562 (44.2)	9,436 (49.1)
Unemployed	3,211 (23.9)	1,309 (29.1)	421 (33.1)	4,941 (25.7)
Unemployed but currently studying	3,434 (25.5)	1,112 (24.8)	288 (22.7)	4,834 (25.2)
Ignored/Missing				2,615 (0.1)^a^
Per capita income				
No income	1,682 (11.1)	577 (11.5)	173 (12.0)	2,432 (11.3)
0–0.25 minimum wage	8,580 (56.8)	2,428 (48.3)	681 (47.2)	11,689 (54.2)
0.25–0.5 minimum wage	2,440 (16.2)	835 (16.6)	241 (16.7)	3,516 (16.3)
0.5–1 minimum wage	1,910 (12.7)	961 (19.1)	285 (19.7)	3,156 (14.6)
> 1 minimum wage	482 (3.2)	225 (4.5)	64 (4.4)	771 (3.6)
Ignored/Missing				1 (0.0)^a^
**Household characteristics**				
Region of residence				
North	3,636 (24.1)	1,164 (23.2)	298 (20.6)	5,098 (23.6)
Northeast	6,401 (42.4)	1,822 (36.2)	497 (34.4)	8,720 (40.4)
Southeast	1,966 (13.2)	814 (16.2)	297 (20.6)	3,107 (14.4)
South	346 (2.3)	202 (4.0)	88 (6.1)	636 (3.0)
Midwest	2,716 (18.0)	1,024 (20.4)	264 (18.3)	4,004 (18.6)
Area of residence				
Urban	12,100 (80.2)	3,894 (77.6)	1,128 (78.3)	17,122 (79.5)
Rural	2,984 (19.8)	1,125 (22.4)	312 (21.7)	4,421 (20.5)
Ignored/Missing				22 (0.0)^a^
Household density				
≤ 0.5 inhab/room	5,014 (33.7)	2,141 (43.2)	619 (43.7)	7,774 (36.6)
0.5–0.75 inhab/room	2,802 (18.8)	848 (17.1)	230 (16.2)	3,880 (18.2)
0.75–1.00 inhab/room	3,320 (22.3)	970 (19.6)	253 (17.8)	4,543 (21.4)
> 1.00 inhab/room	3,754 (25.2)	995 (20.1)	316 (22.3)	5,065 (23.8)
Ignored/Missing				338 (0.01)
Construction material				
Bricks/Cement	10,429 (70.0)	3,347 (67.5)	975 (68.7)	14,751 (69.3)
Wood/Taipa/Others	4,473 (30.0)	1,610 (32.5)	444 (31.3)	6,527 (30.7)
Ignored/Missing				318 (0.01)^a^
Water supply				
Public network (tap water)	10,171 (68.3)	3,311 (66.8)	978 (68.9)	14,460 (68.0)
Well/Natural source/Others	4,731 (31.7)	1,646 (33.2)	441 (31.1)	6,818 (32.0)
Ignored/Missing				318 (0.01)^a^
Electricity				
Electricity with counter	13,566 (91.0)	4,468 (90.1)	1,278 (90.1)	19,312 (90.8)
Electricity without counter	1,336 (9.0)	489 (9.9)	141 (9.9)	1,966 (9.2)
Ignored/Missing				318 (0.01)^a^
Waste disposal system				
Public network/Septic tank	4,823 (32.8)	1,588 (32.6)	506 (36.5)	6,917 (33.0)
Homemade tank/Ditch/Others	9,864 (67.2)	3,285 (67.4)	879 (63.5)	14,028 (67.0)
Ignored/Missing				705 (0.03)^a^
Garbage disposal				
Public collection system	11,409 (76.6)	3,654 (73.7)	1,070 (75.4)	16,133 (75.8)
Burned/Buried/Outdoor disposal/Others	3,494 (23.4)	1,303 (26.3)	349 (24.6)	5,146 (24.2)
Ignored/Missing				317 (0.01)^a^
High-burden cluster municipality				
No	5,148 (34.1)	1,987 (39.5)	679 (47.0)	7,814 (36.2)
Yes	9,947 (65.9)	3,039 (60.5)	765 (53.0)	13,751 (63.8)
**Clinical characteristics**				
WHO operational classification				
Paucibacillary	7,698 (51.0)	968 (19.3)	128 (8.9)	8,794 (40.8)
Multibacillary	7,396 (49.0)	4,058 (80.7)	1,316 (91.1)	12,770 (59.2)
Ignored/Missing				3 (0.0)^a^
Presence of lesions				
No	9,123 (60.4)	1,862 (37.0)	432 (29.9)	12,419 (51.9)
Yes	5,940 (39.4)	3,135 (62.4)	1,004 (69.5)	11,419 (47.8)
Ignored/Missing	32 (0.2)	29 (0.6)	8 (0.6)	73 (0.3)^a^

^a^The percentage of ignored/missing data refers a part of the total

In multivariate analysis, the odds of G1D were higher among leprosy cases aged over 15 years (OR 2.39; 95%CI 2.06–2.77), as well as among those with lower levels of education – no education/pre-school (OR 1.64; 95%CI 1.40–1.93), 1–5 years of education (OR 1.48; 95%CI 1.28–1.70), and 6–9 years of education (OR 1.28; 95%CI 1.10–1.48), unemployed (OR 1.19; 95%CI 1.06–1.32) and living in rural areas (OR 1.14; 95%CI 1.04–1.26) (Table 2). Cases with MB leprosy (OR 3.50; 95%CI 3.13–3.92) and with lesions (OR 1.12; 95%CI 1.02–1.24) were also more likely to have G1D. Factors that showed to be protective against G1D included being female (OR 0.81; 95%CI 0.75–0.88), increased household density (> 1 inhab/romm – OR 0.79; 95%CI 0.71–0.88) and living in a high-incidence cluster municipality (OR 0.85; 95%CI 0.78–0.93) (Table 2).

**Table 2:** Univariate and adjusted analyses for grade of physical disabilities. The 100 Million Brazilian Cohort, 2007–2014

	Grade 1		Grade 2	
	OR_**crude**_^a^ (95%CI)	OR_**adj**_^b**,**c^ (95%CI)	OR_**crude**_^a^ (95%CI)	OR_**adj**_^b**,**c^ (95%CI)
	(N = 5,026)	(N = 5,026)	(N = 1,444)	(N = 1,444)
**Individual characteristics**				
Age				
Up to 15 years old	1.00	1.00	1.00	1.00
> 15 years old	3.41 (3.01–3.87)	2.39 (2.06–2.77)	3.28 (2.63–4.11)	1.95 (1.51–2.50)
Sex				
Male	1.00	1.00	1.00	1.00
Female	0.62 (0.58–0.66)	0.81 (0.75–0.88)	0.48 (0.38–0.48)	0.61 (0.53–0.70)
Schooling				
No education/Pre-school	1.60 (0.86–2.94)	1.64 (1.40–1.93)	3.83 (0.93–15.88)	1.91 (1.44–2.53)
Primary School (1–5 years)	1.30 (0.71–2.38)	1.48 (1.28–1.70)	2.67 (0.65–11.04)	1.64 (1.27–2.12)
High School (6–9 years)	0.95 (0.52–1.74)	1.28 (1.10–1.48)	1.82 (0.44–7.55)	1.31 (1.00–1.72)
Senior High School (10–12 years)	0.72 (0.39–1.32)	–	1.27 (0.30–5.34)	–
Higher Education (≥12 years)	1.00	1.00	1.00	1.00
Work condition				
Employed	1.00	1.00	1.00	1.00
Unemployed	1.06 (0.98–1.16)	1.19 (1.06–1.32)	1.01 (0.86–1.18)	1.47 (1.23–1.74)
Unemployed but currently studying	1.34 (1.23–1.45)	1.13 (1.03–1.24)	1.59 (1.39–1.81)	1.15 (0.98–1.35)
**Household characteristics**				
Region of residence				
North	0.55 (0.46–0.66)	0.91 (0.72–1.15)	0.32 (0.25–0.42)	0.53 (0.39–0.72)
Northeast	0.49 (0.41–0.58)	0.81 (0.64–1.01)	0.31 (0.24–0.39)	0.53 (0.39–0.71)
Southeast	0.70 (0.58–0.85)	1.06 (0.84–1.33)	0.58 (0.45–0.76)	0.80 (0.59–1.08)
South	1.00	1.00	1.00	1.00
Midwest	0.65 (0.54–0.78)	0.86 (0.68–1.09)	0.38 (0.29–0.49)	0.50 (0.36–0.68)
Area of residence				
Urban	1.00	1.00	1.00	1.00
Rural	1.17 (1.08–1.27)	1.14 (1.04–1.26)	1.12 (0.98–1.28)	1.05 (0.89–1.24)
Household density				
≤ 0.5 inhab/room	1.00	1.00	1.00	1.00
0.5–0.75 inhab/room	0.71 (0.65–0.78)	0.87 (0.78–0.98)	0.66 (0.57–0.78)	0.96 (0.80–1.16)
0.75–1.00 inhab/room	0.68 (0.63–0.75)	0.87 (0.78–0.97)	0.62 (0.53–072)	0.85 (0.71–1.03)
> 1.00 inhab/room	0.62 (0.57–0.68)	0.79 (0.71–0.88)	0.68 (0.59–0.79)	1.04 (0.87–1.23)
High-burden cluster municipality				
No	1.00	1.00	1.00	1.00
Yes	0.79 (0.74–0.85)	0.85 (0.78–0.93)	0.58 (0.52–0.65)	0.67 (0.58–0.78)
**Clinical characteristics**				
WHO operational classification				
Paucibacillary	1.00	1.00	1.00	1.00
Multibacillary	4.36 (4.04–4.71)	3.50 (3.13–3.92)	10.70 (8.90–12.87)	8.22 (6.51–10.38)
Presence of lesions				
No	1.00	1.00	1.00	1.00
Yes	2.56 (2.38–2.76)	1.12 (1.02–1.24)	3.44 (3.15–3.93)	1.03 (0.88–1.20)

^a^Univariate logistic regression model accounting for household cluster

^b^Final model of multinomial logistic regression accounting for household cluster with exclusion of the missing data

^c^For all the tests and for permanence of the variables in the final model was used the significance level of 5%

For G2D, the model showed similar risk factors to the G1D analysis (Table 2). Both age above 15 years old (OR 1.95; 95%CI 1.51–2.50) and lower levels of education appeared to influence the odds of had G2D, specifically, no education/pre school (OR 1.91; 95%CI 1.44–2.53) and 1–5 years of education (OR 1.64; 95%CI 1.27–2.12). Being a MB leprosy case was also a risk factor (OR 8.22; 95%CI 6.51–10.38) to have G2D. However, being female (OR 0.61; 95%CI 0.53–0.70) and living in a high-incidence cluster municipality (OR 0.67; 95%CI 0.58–0.78) decreased the odds of presenting G2D at diagnosis. Protective effects were also observed for living in the North (OR 0.53; 95%CI 0.39–0.72), Northeast (OR 0.53; 95%CI 0.39–0.71) and Midwest regions (OR 0.50; 95%CI 0.36–0.68) (Table 2).

## Discussion

This study investigated factors associated with leprosy-related disability in a large Brazilian patient population of 21,565 new leprosy cases. Our results showed lower odds of having grade 1 or grade 2 physical disabilities associated with being a woman, living in the North, Northeast and Midwest regions or in high-incidence clusters, in urban areas, and increased household crowding. However, new leprosy cases aged over 15 years, with a lower levels of education, unemployed and with multibacillary leprosy had higher chances of presenting grade 1 or grade 2 physical disabilities at diagnosis.

The higher likelihood of leprosy-related disabilities found among those older than 15 years is similar to previous studies. In a hyperendemic area of the Midwest region of Brazil, the estimated risk ratio of G2D was 5.3 times higher among patients aged ≥45 years [16]. In the state of Minas Gerais, a retrospective study showed that age above 15 years was an important risk factor for the development of physical disability in leprosy patients as well [17]. A study of patients from the state of Maranhão showed a progressive increase in the chances of developing physical disability among those older than 15 years, ranging from 3 to 10.4 times higher risk [18]. Considering that the duration of the disease is directly related to age and, given the chronic nature of the disease, increasing age may result in more advanced disabilities [17, 19].

Regarding gender, some studies did not identify an association between gender and level of disability [20–22]. However, other studies reported higher grades of physical disability among male individuals with leprosy [17, 23]. Men are generally more exposed to *M. leprae* and have reduced contact with health care, which may delay diagnosis and increases the risk of developing physical disabilities [24]. For the general population in Brazil, between 2012 and 2016, the detection rate of new cases with physical disability grade 2 was higher in males. This rate was 15.2 and 6.1 cases per 1 million among men and women, respectively [5]. Cultural factors may explain the difference by gender as women may be more likely to seek health care [18].

Our study also suggests that higher levels of education were negatively associated with the presence of physical disabilities at diagnosis, which is consistent with the literature [16, 17]. Higher education has been shown to be associated with better understanding of the disease and, consequently, better access and utilization of health services. Regular treatment and evaluation, as well as self-care, are aspects that may prevent the worsening of clinical manifestations [17, 25].

The fact that cases from the Northeast and the North regions were less likely to present G2D contrasts with the findings from Freitas and colleagues (2014) [26], which showed greater proportions of G2D in municipalities with higher incidence rates of leprosy. In their work, the explanation presented for this fact was that better surveillance was leading to a higher detection rate. And subsequently, this was leading to more G2D cases that were found by contact tracing. However, the areas with higher endemicity, in general, do not have a better structured surveillance and care system, as they are systematically poorer. The clusters are located in more vulnerable areas.

Therefore, this fact is likely due to a more sensitive health staff and surveillance system to case detection, therefore more capable of detecting leprosy cases earlier. Assuming that disability is a marker for late diagnosis, it is expected that regions of high endemicity will show a lower chance of patients presenting with grades 1 and 2 disability. G2D, as already mentioned, may indicate late diagnosis and a suboptimal surveillance system. According to Penna et al. (2009) [14], access to primary health care units has improved mainly in rural areas and small towns, improving the diagnosis of leprosy in the first decade of this century. Also, as her work emphasizes, there is a cultural component related to the presence of skin lesions in populations that are used to seeing this type of clinical manifestation of the disease (i.e., in highly endemic areas), coupled with health-seeking behavior among these individuals.

The study by Freitas et al. (2014) [26] looked at risk factors and identified a high new case detection rate in the Midwest and North regions compared to the South, large cities and greater urbanization, median and high illiteracy rate, income inequality (Gini index), hosehold’ crowding, worse sanitation condition, and percentage of cases with grade 2 disability. Differences between these findings and our results may stem from the fact that our study focused on G2D at diagnosis, which does not necessarily mirror risk factors for higher incidence or new case detection rate. As we are hypothesizing, high incidence and detection of disabilities may be influenced by different factors. It is technically difficult to separate short- and long-term effects of increased surveillance [8].

The association between the proportion of multibacillary leprosy and presentation of G2D has been shown in the past [16, 27, 28]. Studies conducted in some Brazilian municipalities indicate that at the time of diagnosis, educational level and operational classification are statistically associated with the development of physical disabilities. It is emphasized that multibacillary patients are twice more likely to develop sequelae than paucibacillary patients [29].

Our study has several strengths. Although there are studies addressing leprosy related disabilities, (i) our large sample size and extensive follow up period allowed us to evaluate determinants of leprosy-related physical disabilities to an extent that is rarely possible; (ii) this study linked data from over 100 million individuals and was able to assess factors associated with physical disability in an unprecedented way; (iii) additionally, using administrative databases linkage we also were able to evaluate a wider range of variables available in CadÚnico and; (iv) unlike other studies, we analyzed the most vulnerable fraction of the Brazilian population, for whom biological and poverty-related risk factors for leprosy overlap.

Nevertheless, our study has some limitations. The use of secondary data originated from routine surveillance activities always brings the issue of completeness of information. We did not have complete information on disability evaluation at diagnosis (*n* = 1557) and at discharge. The latter was poorly collected to an extent that did not allow us to use that timepoint in the analysis. Efforts should be undertaken to stress the importance of performing this evaluation at discharge and record it in the information systems. Other factors associated with disability were not available in our database and therefore, could not be assessed, such as health services characteristics and patients’ perception and knowledge about leprosy. In addition, although there is biological plausibility and references showing the association between leprosy reactions and presentation of disability [25, 27], this information was not obtained in our study population at time of diagnosis. Reactions are reported in the system only when the episode happens during the course of treatment, and this precludes us from assessing this topic in our study.

## Conclusion

The findings suggest that the development of leprosy-related physical disabilities remains a public health problem, mainly for those with severe clinical features and worse socioeconomic conditions. Early diagnosis is paramount to decrease the incidence of disabilities and focus should be given to younger patients, considering these individuals are of working age. Our study points to the need for strengthening control actions in non-endemic areas in Brazil, where cases may be missed when presented at early stages in the course of infection. Besides, social protection policies and initiatives are key to lead us to effective leprosy control – evidence that has been put forth a century ago [30] and yet remains valid. Future research should study disability-related socioeconomic and clinical factors at the end of treatment and explore if the findings from this work will hold among relapses or reinfections.

## Data Availability

The data that support the findings of this study are available from Center of Data and Knowledge Integration for Health (Cidacs – https://cidacs.bahia.fiocruz.br/) but restrictions apply to the availability of these data, which were used under license for the current study, and so are not publicly available. Data are however available from the authors upon reasonable request and with permission of Cidacs.

## Abbreviations

95%CI: Confidence Interval of 95%
BBSRC: Biotechnology and Biological Sciences Research Council
CadÚnico: Cadastro Único para Programas Sociais
Cidacs: Centro de Integração de Dados e Conhecimentos para Saúde
CNPq: Conselho Nacional de Desenvolvimento Científico e Tecnológico
CONFAP: Conselho Nacional das Fundações Estaduais de Amparo à Pesquisa
ESRC: Economic and Social Research Council
FAPDF: Fundação de Apoio à Pesquisa do Distrito Federal
G0D: Grade 0 disability
G1D: Grade 1 disability
G2D: Grade 2 disability
M. leprae: Mycobacterium leprae
MB: Multibacillary
MRC: Medical Research Council
OR: Odds Ratio
PB: Paucibacillary
SINAN: Sistema de Informação de Agravos de Notificação
WHO: World Health Organization
